# Risk of Cardiovascular Disease Death in Older Malignant Melanoma Patients: A Population-Based Study

**DOI:** 10.3390/cancers14194783

**Published:** 2022-09-30

**Authors:** Jiapeng Miao, Yujie Wang, Xiaoyu Gu, Wenrui Lin, Zhen Ouyang, Mi Wang, Mingliang Chen, Shuang Zhao, Xianggui Wang, Juan Su

**Affiliations:** 1 Department of Dermatology, Hunan Key Laboratory of Skin Cancer and Psoriasis, Hunan Engineering Research Center of Skin Health and Disease, Xiangya Clinical Research Center for Cancer Immunotherapy, Xiangya Hospital, Central South University, 87 Xiangya Road, Changsha 410008, China; 2 National Engineering Research Center of Personalized Diagnostic and Therapeutic Technology, 87 Xiangya Road, Changsha 410008, China; 3 Department of Cardiovascular Medicine, Xiangya Hospital, Central South University, 87 Xiangya Road, Changsha 410008, China; 4 Eye Center of Xiangya Hospital, Central South University, 87 Xiangya Road, Changsha 410008, China; 5 Hunan Key Laboratory of Ophthalmology, Xiangya Hospital, Central South University, 87 Xiangya Road, Changsha 410008, China

**Keywords:** malignant melanoma, cardio-oncology, cardiovascular disease death

## Abstract

**Simple Summary:**

Studies have shown that the risk of cardiovascular disease (CVD) death is significantly increased in older (65 or older) malignant melanoma (MM) patients. Proportion of death, standardized mortality ratio (SMR) and cumulative mortality were used to compare the differences in mortality between MM and CVD in older MM patients. Our study revealed older MM patients died from diseases other than primary cancer, and CVD was the leading cause. MM patients had a 1.98-fold higher risk of CVD death than the general population, which signals the importance of prevention and treatment of cardiovascular disease in patients with MM.

**Abstract:**

Noncancer deaths account for a large proportion of deaths in patients with malignant melanoma (MM), but the risk of cardiovascular disease (CVD) death in older MM patients remains unclear. This study aimed to estimate the risk of CVD death in older MM patients. Data on older MM patients were obtained in the Surveillance, Epidemiology, and End Results database. Risk of CVD death was calculated by standardized mortality rates (SMRs), cumulative mortality and proportion of different causes of death. MM patients had a higher risk of CVD death than general populations (SMR = 1.98; 95% CI 1.93–2.03, *p* < 0.001). CVD death was more common in MM patients who were diagnosed at age 85 or older, had a localized stage, were white, had surgical treatment, had a primary head/neck/upper limb site and had a low-grade and superficial spreading/lentigo malignant pathologic type. Cumulative CVD mortality was more common than primary cancer in all older age groups, male or female, and patients with localized-stage disease. Other than primary cancer, CVD was the main cause of death in older patients diagnosed with MM. Our findings highlight CVD death is an important competing event of deaths in older MM patients, and more attention should be paid to reducing CVD death to improve survival.

## 1. Introduction

In recent decades, the incidence of malignant melanoma (MM) has increased dramatically and raised numerous health concerns [1,2]. Research has shown that malignancy, including MM, is associated with an increased risk of cardiovascular disease (CVD) [3]. On the other hand, noncancer deaths account for a large percentage of deaths in patients with various malignancies [4,5], and MM is no exception, especially for CVD death [6]. Therefore, identifying and controlling these noncancer factors can improve overall survival and reduce mortality.

Epidemiologically, MM mainly occurs in individuals aged 40 to 60 years [7]. CVD deaths, which most frequently appear in the elderly, should focus attention on cause-specific mortality at older ages with implications for prevention strategies [8]. However, less is known about CVD death in MM patients, because most research has focused on CVD side effects after oncological treatment, making limited contributions to assessing prognosis [9,10,11]. The use of immune checkpoint inhibitors (ICIs) in MM treatment significantly improved the prognosis of cancer, but also brought the risk of death from noncancer diseases such as CVD for MM patients, which was verified by the meta-analysis of Inno A et al. [12]. MM itself increases the risk of death from CVD. One study reported that the risk of CVD death in MM patients older than 65 was 43.309-fold higher than that in patients less than 50 years old [13], which further indicates that attention should be paid to the risk of CVD death in older MM patients. However, there have been few reports of an increased risk of CVD death in this population. Hence, it is still necessary to further identify this risk of CVD death among older MM patients so that prevention and treatment can be carried out according to the different risks of CVD death to achieve precision medicine.

In this study, we conducted a population-based analysis based on the Surveillance, Epidemiology, and End Results (SEER) database to confirm high-risk subgroups causing CVD death in older MM patients.

## 2. Materials and Methods

### 2.1. Data Sources

Data were obtained from the SEER-9 database of the US National Cancer Institute, using SEER*Stat [14]. Data on the general population who died from CVD were downloaded from the Centers for Disease Control and Prevention Wide-Ranging Online Data for Epidemiologic Research (CDC WONDER) [15] for time trend analysis [16]. Data for a standardized cohort reflecting the US general population were also obtained from CDC WONDER [17]. Ethics committee approval and patient informed consent were not required for this study.

### 2.2. Study Population and Data Collection

In this study, we included older patients (65 or older) diagnosed with single MM with a maximum follow-up period of 44 years. We collected data concerning age at diagnosis, gender, race (white, black and others), stage (localized, regional, distant and unknown), surgery (yes/no), grade (grades I, II, III and IV), follow-up time, end events and cause of death. The detailed grade and stage information can be found in the SEER research data record description. The inclusion criteria were as follows: (1) diagnosed with MM as primary cancer; (2) diagnosed between 1975 and 2018; (3) age at diagnosis 65 years or older; (4) clear cause of death. The exclusion criteria were as follows: (1) no active follow-up; (2) other primary cancers; (3) lost to follow-up. Follow-up was in accordance with the SEER database.

### 2.3. Study Design and Outcomes

First, the proportion of each cause of death in each subgroup (categorized by age at diagnosis, gender, race, grade, stage, histology, primary site, follow-up time and surgery) or their combinations was calculated. Cumulative mortality was calculated, and cumulative mortality curves ware generated according to follow-up time. Furthermore, we calculated the standardized mortality ratio (SMR) for CVD in each subgroup (categorized by age at diagnosis, gender and race) of the overall, surgical and nonsurgical cohorts. The primary outcome was death from any cause, including CVD. Causes of death were classified into 10 categories (Table S1) according to the SEER Cause of Death Record [18,19], which is based on the WHO International Classification of Diseases and Related Health Problems (8th, 9th and 10th revisions) [20]. CVD includes aortic aneurysm and dissection; atherosclerosis; cerebrovascular diseases; diseases of heart; hypertension without heart disease; other diseases of arteries, arterioles and capillaries. Follow-up ended at the occurrence of the primary endpoint event or at the end of follow-up (31 December 2018). The study design is shown in Figure S1.

### 2.4. Statistical Analysis

We used Excel software (Microsoft Excel for Mac, version 16.52) to calculate the proportion of each cause of death and to plot the percentage stack histograms. Cumulative mortality and cumulative mortality curves were generated by the competing risk model (CRM) using the “ggplot2,” “survival,” and “cmprsk” packages in R (R for Mac OS X GUI, version 4.1.0). We generated cumulative incidence curves to describe the incidence over time of death from cardiovascular disease, malignant melanoma, other cancers or other noncancer deaths [21].

The SMR for CVD was calculated as the ratio of observed to expected CVD deaths. The observed CVD deaths were the actual deaths overall in MM patients. The expected CVD death tolls were calculated by multiplying the CVD mortality in the general population by person years. The person years was the sum of follow-up time for all MM patients. The 95% confidence interval (95% CI) and *p* value of SMRs were calculated as previously shown [22,23]. The estimates were presented as SMRs and 95% CIs. The effect of a variable in any analysis was considered significant when *p <* 0.05 (See page 2 of the Supplementary Materials for formulas).

SPSS software (SPSS 26.0, SPSS Inc.) was used for statistical testing. It was used to describe the follow-up time, and the Kolmogorov–Smirnov test was used to test whether the follow-up time obeyed a normal distribution. If *p* > 0.05, the data obeyed a normal distribution. If normal distribution was obeyed, the follow-up time was described using mean ± standard deviation; otherwise, it was described using median (interquartile range).

## 3. Results

### 3.1. Characteristics of the Study Population

There were 39,510 patients diagnosed with MM between 1975 and 2018 in the SEER database, of which 21,729 (45.0%) died during the follow-up period, including 7424 (34.2%) CVD deaths. The median follow-up time was 4.17 (1.58, 8.75) years. Half of the patients’ (52.3%) age at diagnosis was between 65 and 74 years. Most patients were male (58.3%), white (96.2%), had localized disease (63.7%), and most of them had had surgery (92.7%). Similarly, patients whose age at diagnosis was between 65 and 74 years (41.2%), were male (58.0%), white (97.5%), had localized disease (67.8%), with low-grade disease (85.4%), and who had had surgery (91.0%) accounted for a higher proportion of deaths overall. A high percentage of patients (90.3%) had low-grade cancer. Patients’ baseline characteristics are shown in Table 1. Characteristics of the general population are shown in Table 2.

### 3.2. Malignant Melanoma Patients’ CVD Risk Compared to the General Population

SMRs of CVD are shown in Table 3. In the overall cohort, MM patients had a significant CVD risk (SMR = 1.98, 95% CI 1.93–2.03). Patients diagnosed at age 65–74 had the largest CVD risk compared to the age-matched general population (SMR = 3.09, 95% CI 2.98–3.21). There was no significant difference in the SMR of CVD between males and females (SMR = 2.00, 95% CI 1.94–2.06 vs. SMR = 1.95, 95% CI 1.88–2.02). The SMR of CVD in white patients was the lowest among all races. The SMRs of CVD in the nonsurgical cohort were higher than those in the corresponding surgical cohort.

### 3.3. Causes of Death in Malignant Melanoma Patients

**Figure 1:**
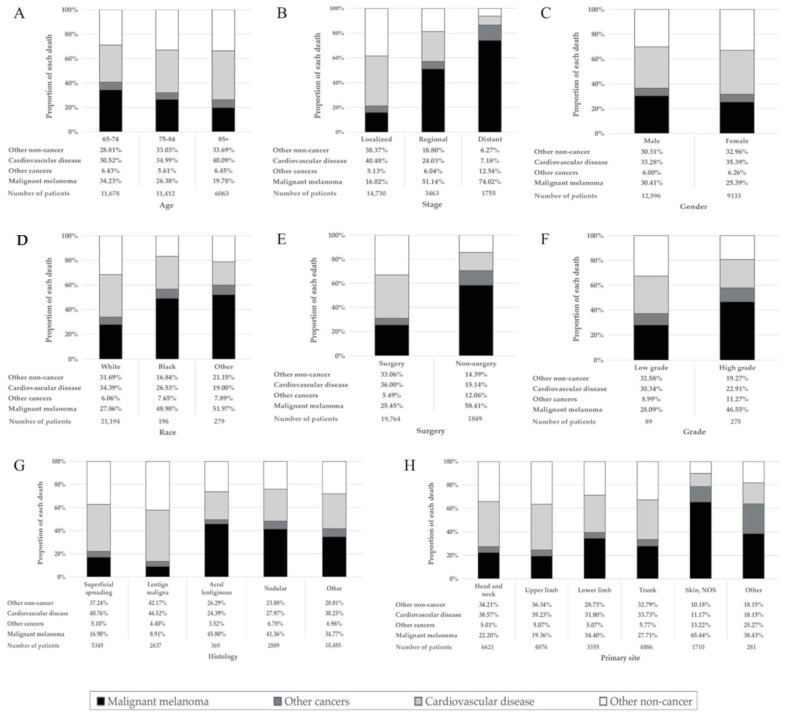
Percentage of each cause of death in different malignant melanoma patient groups. The height of the four differently colored stacked bars shows the different proportions of deaths from malignant melanoma (black), other cancer death (dark gray), cardiovascular disease (light gray) and other noncancer death (white), respectively. (**A**) Patients by different age at diagnosis. (**B**) Patients at different stages. (**C**) Patients of different genders. (**D**) Patients of different races. (**E**) Patients with/without surgical treatment. (**F**) Patients with different grades. (**G**) Patients with different histologies. (**H**) Patients with different primary sites. Abbreviation: NOS, not otherwise specified.

**Figure 2:**
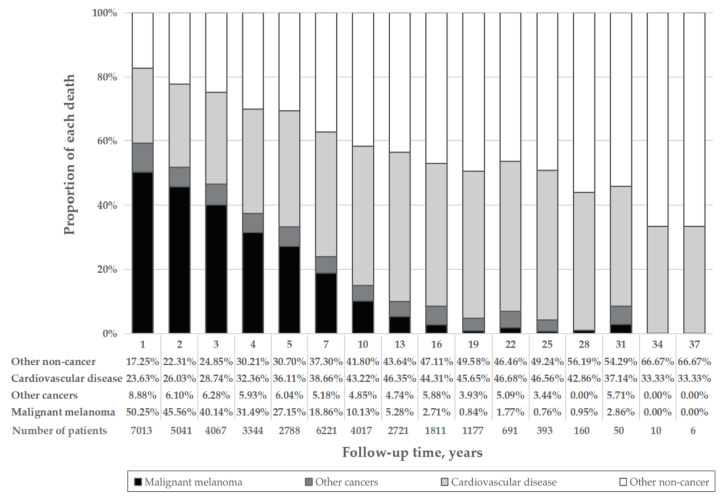
Percentage of each cause of death by follow-up time. The height of the four differently colored stacked bar charts shows the different proportions of deaths from malignant melanoma (black), other cancer death (dark gray), cardiovascular disease (light gray) and other noncancer death (white), respectively. “Years” refers to the middle of the year range of each bar.

Among patients in the overall MM cohort who died, 7424 (34.2%) died due to CVD (Table 1). The proportion of MM death declined, whereas that of CVD deaths rose with age (Figure 1A). The percentage of CVD death was much larger than that of MM deaths among patients diagnosed at age 85 or older (40.09% vs. 19.78%) (Figure 1A), with localized disease (40.48% vs. 16.02%) (Figure 1B) and having had surgery (36.00% vs. 25.45%) (Figure 1E). CVD was the main cause of death in MM patients diagnosed at all ages. The proportion of CVD deaths in the localized disease group was much higher than that in the distant disease group (40.48% vs. 7.18%) (Figure 1B). With the increase in follow-up time, the proportion of CVD deaths increased, whereas that of MM death decreased. Furthermore, CVD death was the main cause of death in patients followed up for longer than 7 years, with a greater proportion of death than that of MM (Figure 2). Proportions by different year of diagnosis are shown in Figure S3.

### 3.4. Subgroup Analysis of Proportion of Deaths

In the subgroup analyses, the percentage of CVD deaths was much higher than MM deaths among patients with localized disease in different age subgroups (Figure S2I), while the distant disease group showed the opposite result (Figure S2K). The proportion of deaths from CVD decreased, and the proportion of deaths from MM increased as MM progressed from a localized stage to a distant stage in patients with or without surgical treatment and had a low or high disease grade (Figure S2A–D). With the increase in age at diagnosis, the proportion of CVD deaths in male and female MM patients showed an increasing trend, while the proportion of MM deaths showed an opposite trend (Figure S2E,F). With the increase in age at diagnosis, the proportion of MM deaths decreased in MM patients with different grades or stages, while the proportion of CVD deaths did not change significantly (Figure S2G,K).

### 3.5. Cumulative Mortality in Malignant Melanoma Patients

**Figure 3:**
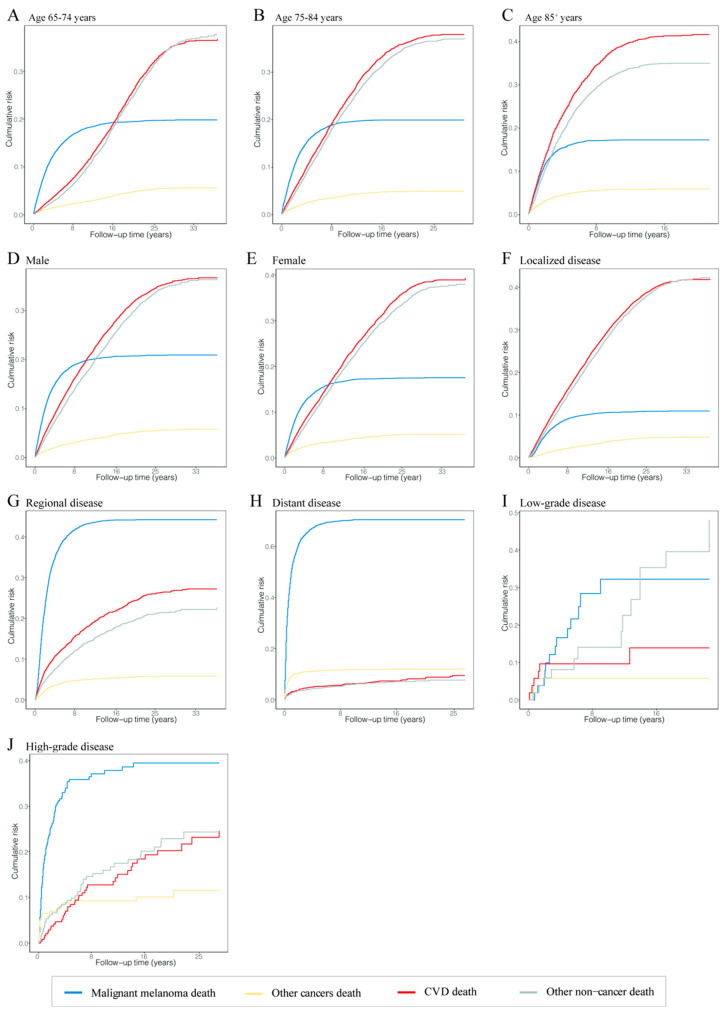
Cumulative mortality curves at time of follow-up in different malignant melanoma patient subgroups. The four curves show the cumulative mortality curves of malignant melanoma death (blue), other cancer death (yellow), cardiovascular disease death (red) and other noncancer death (gray), respectively. (**A**) Patients diagnosed at 65–74 years. (**B**) Patients diagnosed at 75–84 years. (**C**) Patients diagnosed at 85^+^ years. (**D**) Male patients. (**E**) Female patients. (**F**) Patients with localized disease. (**G**) Patients with regional disease. (**H**) Patients with distant disease. (**I**) Patients with low-grade disease. (**J**) Patients with high-grade disease. Abbreviation: CVD, cardiovascular disease.

The risk of death from CVD and other noncancers was the leading risk in patients diagnosed with MM at 65–74 years old, and their risks of death were comparable; the risk of death from CVD increased with age at diagnosis; the risk of death from MM was equal in different age groups at an intermediate level (Figure 3A–C). In terms of disease stages, CVD and other noncancer deaths were the main risks for death in patients with localized disease, while the risk of MM death was increased and the risk of CVD and other noncancer deaths was decreased dramatically in MM patients with localized to distant disease stages (Figure 3F,H). The same trend was shown in patients with different grades (Figure 3I,J).

In different age groups, the cumulative mortality of CVD in patients diagnosed at age 85 or older was higher than at ages 65–74 and 75–84 at 20 years of follow-up (41.65% vs. 25.04% and 36.03%). The cumulative mortality of CVD in patients with localized disease was also higher than in patients with regional and distant disease at 20 years of follow-up (38.91% vs. 26.23% and 9.51%). The cumulative mortalities of CVD were equal in patients with low- and high-grade cancer at 20 years of follow-up (27.05% vs. 26.77%). CVD was the major cause of death in patients aged 85 or older and with localized disease at any follow-up point. The different causes of cumulative mortality overall and in subgroups are shown in Tables S2–S7.

## 4. Discussions

This population-based study shows that noncancer deaths account for a large proportion of deaths in older MM patients, among which CVD is the main cause. CVD death was higher in MM patients who were diagnosed at age 85 or older, had a localized stage, were white, had surgical treatment, had a primary site of head/neck/upper limb and had a low-grade and superficial spreading/lentigo malignant pathologic type. Cumulative CVD mortality was higher in all older age groups, male or female, and patients with localized-stage disease. Moreover, with the extension of follow-up time, CVD became the leading cause of death.

Calculating the proportions of different causes of death [24] can reflect the proportions of causes of death in diverse subgroups of older MM patients. The results of a SEER-based study demonstrated that MM was one of the top 10 cancers with the highest CVD mortality [25]. In a retrospective analysis of 127 older MM patients, 34.7% died of non-MM diseases, including CVD [26]. Similarly, a study of 213,716 MM patients showed that CVD-specific death increased with age at MM diagnosis. CVD-specific death was 6.559-fold higher in MM patients older than 70 years than in patients aged 40 to 69 years [27]. The results of our study are therefore consistent with previous studies. A study of Sturgeon KM et al. found advanced age and comorbidities are risk factors for CVD death, especially malignant comorbidities, including MM [28]. However, this study focused only on the general population, and did not look at specific groups, such as older patients. The overall results may mask the characteristics of the older subgroup. The interaction between CVD and melanoma was not analyzed in a crude analysis of the proportion of deaths in the year of diagnosis.

Our study compared not only the age at diagnosis but also analyzed the proportion of deaths in different subgroups. Furthermore, we used the CRM to evaluate the risks of diverse causes of death, calculate cumulative mortality and plot cumulative mortality curves [29]. Our findings are partially similar to those of a previous SEER-based study that showed that among MM patients diagnosed at different ages, patients diagnosed at age 65 and older had the highest CVD mortality [13]. However, that study only analyzed the overall risk of death in older MM patients. In our study, older MM patients were the research object and were divided into different subgroups for the study of CVD-specific mortality. By subgroup analysis, we could further identify subgroups with higher CVD-specific mortality in older MM patients to better prevent the occurrence of CVD events in these subgroups. The study of Stolzfus KC et al. also explored the risk of CVD death in the overall MM population, and analyzed the risk factors for CVD using Cox regression [30]. However, this study did not explore specific populations, such as the elderly, and our CRM is more suitable for the analysis of CVD mortality competitive risk events.

It is a natural rule that the overall mortality risk, including CVD, rises with increasing age. However, contrary to this rule, our study found that the SMRs for CVD were higher in the 65–74 and 75–84 age groups than in the 85 or older group. Because patients in these two age groups are younger than individuals in the 85 or older group, they will be more likely to benefit from stronger cardiovascular risk factor monitoring and modification, as well as surveillance for cancer-treatment-related cardiotoxicity.

Our results show that females have a lower overall mortality risk than males, which is consistent with several previous studies [31,32], but there was no significant difference in the proportion of causes of death between males and females. Joosse A et al.’s study showed that females may have more antioxidant enzymes and different immune responses, which may have an impact on mortality [33].

As surgery is the primary treatment for MM [34], our results show that the SMR of CVD in MM patients in the nonsurgical cohort was higher than that in the surgical cohort. However, the number of patients in the nonsurgical cohort was significantly lower than that in the surgical cohort, and this conclusion needs to be further confirmed by increasing the number of those in the nonsurgical cohort. Those MM patients who underwent surgical treatment were also protected from the cardiovascular toxicity of anticancer drug therapy. Although data demonstrate a better outcome with surgical resection, this has only been observed in early-stage melanoma, and unresectable metastatic melanoma is usually treated systemically, such as targeted drug therapy, combination chemotherapy and immunotherapy. Currently, systemic therapy is also one of the main treatment modalities for MM. Systemic anticancer therapy inevitably brings about systemic adverse effects, including the cardiovascular system. It has been shown that anti-PD1 therapy can cause disruption of cardiac immune homeostasis and lead to myocardial impairment, and blockading tumor necrosis factor α may be a way to prevent antitumor-therapy-related cardiotoxicity, which is known as an immune-related adverse event (irAE) [35]. It has been demonstrated that some antitumor monoclonal antibodies may cause myocardial damage through an inflammation-dependent pathway, which in turn may cause CVD. ICIs in monotherapy or in combination could increase the risk of fatal myocarditis [36,37]. BRAF and MEK inhibitors and ICIs have been utilized as first-line therapies for patients with metastatic MM, with durable efficacy responses and, in some cases, curing individuals. However, various side effects may occur, in particular, an increase in overall morbidity and mortality from cardiovascular toxicity, with toxicity ranging from asymptomatic QT prolongation and mild left ventricular dysfunction to fulminant myocarditis and potentially life-threatening arrhythmias [38]. The development of myocarditis induced by ICIs is based on the presence of co-expressed molecules between melanoma cells and cardiomyocytes [39]. Another study reported immune-related cardiotoxicity after ipilimumab/nivolumab/pembrolizumab in the treatment of MM, manifesting as heart failure, cardiomyopathy, conduction block, myocardial fibrosis and myocarditis, which is the largest case series describing cardiotoxicity of ICIs to date [40]. It is evident that MM patients on systemic therapy may be complicated by irAEs, leading to myocardial damage, which may be life-threatening in severe cases.

Cytokines can regulate tumor growth by modulating the immune system, and directly inhibit tumor cell growth by inhibiting tumor cell proliferation and promoting tumor cell apoptosis. To date, two cytokines, high-dose IL-2 (HD-IL-2) and IFN-α, have been approved by the FDA for the treatment of MM [41]. In addition, the expression of specific tumor cytokines is an important biomarker of immunotherapeutic response in melanoma [42]. However, some cytokines have dual effects on tumors; IL-6 and IL-8 can directly suppress MM cells or recruit immunosuppressive cells to the tumor microenvironment or promote tumor angiogenesis, such as myeloid-derived suppressor cells (MDSCs), regulatory T cells (T regs) and tumor-associated macrophages (TAMs). In contrast, IL-6 has anticancer effects in the early stages of MM [43]. In addition, it was shown that IL-1 can promote MM metastasis through NF-κB pathway activation of endothelial cells, while the use of the IL-1 receptor antagonist reduced lung metastasis in mice with B16 melanoma. Thus, cytokines can have different effects on MM depending on their type or MM stage [44].

As stated in the conclusion, among the factors affecting the CVD mortality in MM patients, we cannot account for the uncontrollable factors, such as gender, age of diagnosis, race, grade, pathological type and primary site. However, for other factors, they can be considered controllable. For MM patients, we know that the lower the age of diagnosis, the higher the CVD mortality. Therefore, these patients should pay more attention to the treatment and prevention of noncancer diseases such as CVD while treating primary MM. In addition, it can be seen from Figure 3F–H that the cumulative risk of death from all causes was low in the early follow-up of localized MM patients (with a low slope of all curves). However, the cumulative risk of death from CVD was maximized in the short-term follow-up of regional and distant MM patients (the CVD curve showed a steep increase). This suggests that once the disease progresses from the localized stage to the regional or distant stage, the patient is likely to die from CVD at an early stage. Therefore, it is necessary to pay more attention to regular screening, early detection and early treatment of MM to avoid disease progression as far as possible to reduce CVD mortality. For those MM patients with uncontrollable factors, we should pay more attention to them. The existence of these factors indicates that the CVD mortality is high and unavoidable, so we can only strengthen CVD surveillance and treatment to reduce the CVD mortality as much as possible.

Our study has some limitations that should be noted. Firstly, the SEER database does not include information on patients’ comorbidities, and there are no basic CVD data on MM patients who died of CVD, although this study took the general population as a control and included CVD patients, which reduced the interference of comorbidities to some extent. Secondly, we found only some factors that may be associated with CVD death in MM patients. Due to the lack of CVD-related risk factors in the database, we could not explore the impact of these risk factors on the results. Nevertheless, our study was designed to identify people at high risk of death from CVD and did not involve risk factor studies. Thirdly, the impact of systemic treatment on CVD cannot be assessed because no data on systemic anticancer treatment are available in the SEER database and cause of death data in general. Finally, there may be some selection bias between the surgical and nonsurgical cohorts in the SEER database in terms of surgical treatment modality, which needs to be further explored.

## 5. Conclusions

Noncancer death accounts for the majority of deaths in older MM patients. Compared to the general population, MM patients have an increased risk of CVD. These results suggest the importance of monitoring cardiovascular comorbidities in MM patients.

## Figures and Tables

**Table 1:** Characteristics of the study population.

Characteristic	No. of MM Patient (% in the Overall Cohort)	No. of Deaths (% in the Overall Deaths)	No. Alive (% in the Overall Alive)
All patients	39,510	21,729	17,781
Cause of death			
Malignant Melanoma	NA	6149 (28.3)	NA
Other cancers	NA	1328 (6.1)	NA
CVD	NA	7424 (34.2)	NA
Other noncancer ^a^	NA	6828 (31.4)	NA
Diagnostic age (y)			
65–74	20,674 (52.3)	8947 (41.2)	11,727 (66.0)
75–84	13,229 (33.5)	8454 (38.9)	4775 (26.8)
85^+^	5607 (14.2)	4328 (19.9)	1279 (7.2)
Gender			
Male	23,030 (58.3)	12,596 (58.0)	10,434 (58.7)
Female	16480 (41.7)	9133 (42.0)	7347 (41.3)
Race			
White	37,993 (96.2)	21,194 (97.5)	16,799 (94.5)
Black	246 (0.6)	196 (0.9)	50 (0.3)
Other ^b^	449 (1.2)	279 (1.3)	170 (1.0)
Unknown	822 (2.0)	60 (0.3)	762 (4.2)
Stage			
Localized	25,182 (63.7)	14,730 (67.8)	10,452 (58.8)
Regional	4143 (10.5)	3463 (15.9)	680 (3.8)
Distant	1861 (4.7)	1755 (7.1)	106 (0.6)
Unknown	8324 (21.1)	1781 (8.2)	6543 (36.8)
Grade			
Low (Grade I+II)	35,667 (90.3)	18,563 (85.4)	17,104 (96.2)
High (Grade III+IV)	3843 (9.7)	3166 (14.6)	677 (3.8)
Surgery			
Yes	36,632 (92.7)	19,764 (91.0)	16,868 (94.9)
No	2622 (6.6)	1849 (8.5)	773 (4.3)
Unknown	256 (0.7)	116 (0.5)	140 (0.8)

Abbreviations: NA, not applicable; MM, malignant melanoma; CVD, cardiovascular disease. ^a^ “Other non-cancer” refers to infection; endocrine, nervous, respiratory, digestive and kidney diseases; suicide, accidents and homicide; and others. ^b^ “Other” refers to American Indian/Alaska Native, Asian/Pacific Islander and unknown. 85^+^ refers to age 85 or older.

**Table 2:** Characteristics of the general population.

Characteristic	No. of General Population (% in the General Population)	No. of Deaths (% in the Overall Deaths)	No. Alive (% in the Overall Alive)
All populations	40,267,984	631,573	39,636,411
Age (y)			
65–74	21,713,429 (53.9)	114,483 (18.1)	21,598,946 (54.5)
75–84	13,061,122 (32.4)	205,180 (32.5)	12,855,942 (32.4)
85^+^	5,493,433 (13.6)	311,910 (49.4)	5,181,523 (13.1)
Gender			
Male	17,362,960 (43.1)	282,694 (44.8)	17,080,266 (43.1)
Female	22,905,024 (56.9)	348,879 (55.2)	22,556,145 (56.9)
Race			
White	34,971,197 (86.8)	556,057 (88.0)	34,415,140 (86.8)
Black	3,541,901 (8.8)	60,048 (9.5)	3,481,853 (8.8)
Other ^a^	1,754,886 (4.4)	15,468 (2.5)	1,739,418 (4.4)

^a^ “Other” refers to American Indian/Alaska Native, Asian/Pacific Islander and unknown. 85^+^ refers to age 85 or older.

**Table 3:** Cardiovascular-disease-death-related standardized mortality ratios in malignant melanoma patients.

Characteristic	Overall Cohort		Surgery Cohort		Nonsurgery Cohort	
	SMR (95% CI)	*p* Value ^a^	SMR (95% CI)	*p* Value ^a^	SMR (95% CI)	*p* Value ^a^
Overall	1.98 (1.93–2.03)	˂0.001	1.98 (1.93–2.03)	˂0.001	2.47 (2.19–2.78)	˂ 0.001
Diagnostic age (y)						
65–74	3.09 (2.98–3.21)	˂0.001	3.09 (2.98–3.21)	˂0.001	4.61 (3.69–5.70)	˂0.001
75–84	1.30 (1.25–1.34)	˂0.001	1.29 (1.24–1.34)	˂0.001	2.36 (1.92–2.87)	˂0.001
85^+^	1.27 (1.21–1.33)	˂0.001	1.25 (1.19–1.32)	˂0.001	1.72 (1.39–2.11)	˂0.001
Gender						
Male	2.00 (1.94–2.06)	˂0.001	1.99 (1.93–2.05)	˂0.001	2.31 (1.96–2.70)	˂0.001
Female	1.95 (1.88–2.02)	˂0.001	1.93 (1.87–2.00)	˂0.001	2.66 (2.21–3.17)	˂0.001
Race						
White	1.99 (1.94–2.03)	˂0.001	1.97 (1.92–2.02)	˂0.001	2.54 (2.25–2.86)	˂0.001
Black	2.77 (2.07–3.63)	˂0.001	2.87 (2.13–3.78)	˂0.001	2.23 (0.25–8.06)	0.370
Other ^b^	3.13 (2.35–4.10)	˂0.001	3.11 (2.31–4.10)	˂0.001	3.75 (0.75–10.97)	0.052

Abbreviations: SMR, standardized mortality ratio; CI, confidence interval. ^a^ Statistical significance was defined as *p* ˂ 0.05. ^b^ “Other” refers to American Indian/Alaska Native, Asian/Pacific Islander and unknown. 85^+^ refers to age 85 or older.

## Data Availability

All supporting data in the study are publicly available from the SEER database (http://seer.cancer.gov/ (accessed on 23 June 2022)) and CDC WONDER (https://wonder.cdc.gov/ (accessed on 23 June 2022)).

## Supplementary Materials

The following supporting information can be downloaded at: https://www.mdpi.com/article/10.3390/cancers14194783/s1. Figure S1: Study design; Figure S2: Percentage of each cause of death in different malignant melanoma patient subgroups; Figure S3: Percentage of each cause of death in different years of diagnosis; Table S1: Detailed disease clarification of each cause of death; Table S2: Cardiovascular-disease-death-related standardized mortality ratios for different follow-up times; Table S3: Cumulative mortality at follow-up time in patients overall; Table S4: Cumulative mortality at follow-up time in patients diagnosed at age 85 or later; Table S5: Cumulative mortality at follow-up time in patients with localized disease; Table S6: Cumulative mortality at follow-up time in patients with low-grade disease; Table S7: Cumulative CVD mortality at follow-up time in different subgroups.

## References

[1] Welch H.G., Mazer B.L., Adamson A.S. (2021). The Rapid Rise in Cutaneous Melanoma Diagnoses. N. Engl. J. Med..

[2] Siegel R.L., Miller K.D., Fuchs H.E., Jemal A. (2021). Cancer Statistics, 2021. CA Cancer J. Clin..

[3] Strongman H., Gadd S., Matthews A., Mansfield K.E., Stanway S., Lyon A.R., Dos-Santos-Silva I., Smeeth L., Bhaskaran K. (2019). Medium and long-term risks of specific cardiovascular diseases in survivors of 20 adult cancers: A population-based cohort study using multiple linked UK electronic health records databases. Lancet.

[4] Horn S., Stoltzfus K., Mackley H., Lehrer E., Zhou S., Dandekar S., Fox E., Rizk E., Trifiletti D., Rao P. (2020). Long-term causes of death among pediatric patients with cancer. Cancer.

[5] Zaorsky N., Churilla T., Egleston B., Fisher S., Ridge J., Horwitz E., Meyer J. (2017). Causes of death among cancer patients. Ann. Oncol. Off. J. Eur. Soc. Med. Oncol..

[6] Papadopoulos F. (2012). Suicide and cardiovascular death after a cancer diagnosis. N. Engl. J. Med..

[7] Schadendorf D., Fisher D.E., Garbe C., Gershenwald J.E., Grob J.J., Halpern A., Herlyn M., Marchetti M.A., McArthur G., Ribas A. (2015). Melanoma. Nat. Rev. Dis. Primers.

[8] Ijaz N., Buta B., Xue Q.L., Mohess D.T., Bushan A., Tran H., Batchelor W., deFilippi C.R., Walston J.D., Bandeen-Roche K. (2022). Interventions for Frailty Among Older Adults With Cardiovascular Disease: JACC State-of-the-Art Review. J. Am. Coll. Cardiol..

[9] Bronte E., Bronte G., Novo G., Rinaldi G., Bronte F., Passiglia F., Russo A. (2018). Cardiotoxicity mechanisms of the combination of BRAF-inhibitors and MEK-inhibitors. Pharmacol. Ther..

[10] Salem J.E., Manouchehri A., Moey M., Lebrun-Vignes B., Bastarache L., Pariente A., Gobert A., Spano J.P., Balko J.M., Bonaca M.P. (2018). Cardiovascular toxicities associated with immune checkpoint inhibitors: An observational, retrospective, pharmacovigilance study. Lancet Oncol..

[11] Brahmer J.R., Lacchetti C., Schneider B.J., Atkins M.B., Brassil K.J., Caterino J.M., Chau I., Ernstoff M.S., Gardner J.M., Ginex P. (2018). Management of Immune-Related Adverse Events in Patients Treated With Immune Checkpoint Inhibitor Therapy: American Society of Clinical Oncology Clinical Practice Guideline. J. Clin. Oncol..

[12] Inno A., Maurea N., Metro G., Carbone A., Russo A., Gori S. (2021). Immune checkpoint inhibitors-associated pericardial disease: A systematic review of case reports. Cancer Immunol. Immunother..

[13] Elshanbary A., Zaazouee M., Abdelmonem M., Mohammed Y., Abdel-Aziz W. (2021). Risk factors for cardiovascular mortality and melanoma-specific mortality among patients with melanoma: A SEER based study. Eur. J. Cancer Prev. Off. J. Eur. Cancer Prev. Organ. (ECP).

[14] National Cancer Institute Surveillance, Epidemiology, and End Results Program SEER Data & Software. https://seer.cancer.gov/data-software/.

[15] Centers for Disease Control and Prevention About Multiple Cause of Death, 1999–2020. https://wonder.cdc.gov/mcd-icd10.html.

[16] Weberpals J., Jansen L., Muller O.J., Brenner H. (2018). Long-term heart-specific mortality among 347 476 breast cancer patients treated with radiotherapy or chemotherapy: A registry-based cohort study. Eur. Heart J..

[17] Nam H.S., Kim H.C., Kim Y.D., Lee H.S., Kim J., Lee D.H., Heo J.H. (2012). Long-term mortality in patients with stroke of undetermined etiology. Stroke.

[18] National Cancer Institute Surveillance, Epidemiology, and End Results Program SEER Research Plus Data Description Cases Diagnosed in 1975–2018. https://seer.cancer.gov/data-software/documentation/seerstat/nov2020/TextData.FileDescription.pdf.

[19] National Cancer Institute Surveillance, Epidemiology, and End Results Program Dictionary of SEER*Stat Variables. https://seer.cancer.gov/data-software/documentation/seerstat/nov2020/seerstat-variable-dictionary-nov2020.pdf.

[20] National Cancer Institute Surveillance, Epidemiology, and End Results Program SEER Cause of Death Recode 1969+ (03/01/2018) SEER Data Reporting Tools. https://seer.cancer.gov/codrecode/1969_d03012018/index.html.

[21] Abdel-Qadir H., Austin P.C., Lee D.S., Amir E., Tu J.V., Thavendiranathan P., Fung K., Anderson G.M. (2017). A Population-Based Study of Cardiovascular Mortality Following Early-Stage Breast Cancer. JAMA Cardiol..

[22] Altman D.G., Bland J.M. (2011). How to obtain the P value from a confidence interval. BMJ.

[23] Breslow N.E., Day N.E., Heseltine E. (1987). Statistical Methods in Cancer Research. Volume II—The Design and Analysis of Cohort Studies.

[24] Estève J., Benhamou E., Raymond L. (1994). Statistical Methods in Cancer Research. Volume IV. Descriptive Epidemiology.

[25] Sturgeon K.M., Deng L., Bluethmann S.M., Zhou S., Trifiletti D.M., Jiang C., Kelly S.P., Zaorsky N.G. (2019). A population-based study of cardiovascular disease mortality risk in US cancer patients. Eur. Heart J..

[26] Rees M., Liao H., Spillane J., Speakman D., McCormack C., Donahoe S., Pohl M., Webb A., Gyorki D., Henderson M. (2018). Melanoma in the very elderly, management in patients 85years of age and over. J. Geriatr. Oncol..

[27] Abdel-Rahman O. (2017). Risk of cardiac death among cancer survivors in the United States: A SEER database analysis. Expert Rev. Anticancer Ther..

[28] Weilandt J., Diehl K., Schaarschmidt M.L., Kiecker F., Sasama B., Pronk M., Ohletz J., Konnecke A., Muller V., Utikal J. (2021). Patient preferences for treatment of advanced melanoma: Impact of comorbidities. J. Dtsch. Dermatol. Ges..

[29] Lau B., Cole S., Gange S. (2009). Competing risk regression models for epidemiologic data. Am. J. Epidemiol..

[30] Stoltzfus K.C., Zhang Y., Sturgeon K., Sinoway L.I., Trifiletti D.M., Chinchilli V.M., Zaorsky N.G. (2020). Fatal heart disease among cancer patients. Nat. Commun..

[31] Crocetti E., Fancelli L., Manneschi G., Caldarella A., Pimpinelli N., Chiarugi A., Nardini P., Buzzoni C. (2016). Melanoma survival: Sex does matter, but we do not know how. Eur. J. Cancer Prev..

[32] Kindem S., Garcias-Ladaria J., Requena C., Guillen C., Oliver V., Nagore E. (2015). Survival advantage of women in localized melanoma mainly relies on clinical-pathological differences by sex. A retrospective study of 1607 patients in Valencia, Spain. Eur. J. Dermatol..

[33] Joosse A., De Vries E., van Eijck C.H., Eggermont A.M., Nijsten T., Coebergh J.W. (2010). Reactive oxygen species and melanoma: An explanation for gender differences in survival?. Pigment Cell Melanoma Res..

[34] Davis L.E., Shalin S.C., Tackett A.J. (2019). Current state of melanoma diagnosis and treatment. Cancer Biol. Ther..

[35] Michel L., Helfrich I., Hendgen-Cotta U.B., Mincu R.I., Korste S., Mrotzek S.M., Spomer A., Odersky A., Rischpler C., Herrmann K. (2022). Targeting early stages of cardiotoxicity from anti-PD1 immune checkpoint inhibitor therapy. Eur. Heart J..

[36] Quagliariello V., Passariello M., Coppola C., Rea D., Barbieri A., Scherillo M., Monti M.G., Iaffaioli R.V., De Laurentiis M., Ascierto P.A. (2019). Cardiotoxicity and pro-inflammatory effects of the immune checkpoint inhibitor Pembrolizumab associated to Trastuzumab. Int. J. Cardiol..

[37] Quagliariello V., Passariello M., Rea D., Barbieri A., Iovine M., Bonelli A., Caronna A., Botti G., De Lorenzo C., Maurea N. (2020). Evidences of CTLA-4 and PD-1 Blocking Agents-Induced Cardiotoxicity in Cellular and Preclinical Models. J. Pers. Med..

[38] Mukunda N., Vallabhaneni S., Lefebvre B., Fradley M.G. (2022). Cardiotoxicity of Systemic Melanoma Treatments. Curr. Treat. Opt. Oncol..

[39] Arangalage D., Degrauwe N., Michielin O., Monney P., Ozdemir B.C. (2021). Pathophysiology, diagnosis and management of cardiac toxicity induced by immune checkpoint inhibitors and BRAF and MEK inhibitors. Cancer Treat. Rev..

[40] Heinzerling L., Ott P.A., Hodi F.S., Husain A.N., Tajmir-Riahi A., Tawbi H., Pauschinger M., Gajewski T.F., Lipson E.J., Luke J.J. (2016). Cardiotoxicity associated with CTLA4 and PD1 blocking immunotherapy. J. Immunother. Cancer.

[41] Bentebibel S.E., Diab A. (2021). Cytokines in the Treatment of Melanoma. Curr. Oncol. Rep..

[42] Fang S., Xu T., Xiong M., Zhou X., Wang Y., Haydu L.E., Ross M.I., Gershenwald J.E., Prieto V.G., Cormier J.N. (2019). Role of Immune Response, Inflammation, and Tumor Immune Response-Related Cytokines/Chemokines in Melanoma Progression. J. Investig. Dermatol..

[43] Tobin R.P., Jordan K.R., Kapoor P., Spongberg E., Davis D., Vorwald V.M., Couts K.L., Gao D., Smith D.E., Borgers J.S.W. (2019). IL-6 and IL-8 Are Linked with Myeloid-Derived Suppressor Cell Accumulation and Correlate with Poor Clinical Outcomes in Melanoma Patients. Front. Oncol..

[44] Lavi G., Voronov E., Dinarello C.A., Apte R.N., Cohen S. (2007). Sustained delivery of IL-1 Ra from biodegradable microspheres reduces the number of murine B16 melanoma lung metastases. J. Control. Release.

