# Supplementary material for: Risk of Cardiovascular Disease Death in Older Malignant Melanoma Patients: A Population-Based Study
Source: Cancers (Basel). 2022 Sep 30;14(19):4783. doi: 10.3390/cancers14194783 (PMC9563114; doi:10.3390/cancers14194783)
Supplement: Supplementary file 1 [file cancers-14-04783-s001.zip › cancers-1894841-supplementary.pdf]

Supplementary materials

Details on the CDC WONDER data: The detailed information is shown at https://wonder.cdc.gov/wonder/help/mcd.html#.

Mortality data: The mortality data are based on information from all death certificates filed in the fifty states and the District of Columbia.

Cause-of-death: The underlying cause-of-death is defined by the World Health Organization (WHO) as "the disease or injury which initiated the train of events leading directly to death, or the circumstances of the accident or violence which produced the fatal injury." Underlying cause-of-death is selected from the conditions entered by the physician on the cause of death section of the death certificate.

Race: Race and Hispanic origin are reported separately on the death certificate in accordance with standards set forth by the Office of Management and Budget. The American Indian or Alaska Native race category includes: North, Central, and South American Indians, Eskimos, and Aleuts. The Asian or Pacific Islander race category includes Chinese, Filipino, Hawaiian, Japanese, and Other Asian or Pacific Islanders.

Population data: The population estimates are U.S. Census Bureau estimates of U.S. national, state, and county resident populations.

Variables:

(1) Location: HHs Regions, Census Regions and Census Divisions, State, County

(2) Age Groups: 10-year age groups, 5-year age groups, single-year age groups and infant age groups

(3) Race: American Indian or Alaskan Native, Asian / Pacific Islander, Black or African American, White (archive data for years 1999-2004: Black or African American, White, Other Race)

(4) Hispanic Origin: Hispanic or Latino, Not Hispanic or Latino, Not stated (Hispanic Origin not available in archive data 1999-2004)

(5) Gender (Sex): Female, Male

(6) Year of death: 1999-2020 (Year limited in archive data: 1999-2004, or 2005-2006)

(7) Month of death: January through December (Month not available in archive data)

(8) Weekday of death: Sunday through Saturday, Unknown (Weekday not available in archive data)

(9) Autopsy performed: No, Yes, Unknown (Autopsy not available in archive data)

(10) Place of Death: Medical Facility - Inpatient, Medical Facility - Outpatient or ER, Medical Facility - Dead on Arrival, Medical Facility - Status unknown (years 1999-2002 only), Decedent's home, Hospice Facility (years 2003 and later only), Nursing home/long term care, Other, Place of death unknown (Place of Death not available in archive data)

(11) Cause of Death: underlying and multiple causes of death - ICD-10 codes, 113 Selected Causes, 130 Selected Causes (for infants), Drug/Alcohol Induced Causes (not available in archive data), Injury Intent and Mechanism groups (not available in archive data)

(12) Urbanization: classifies population density and other factors at the county level - pick between the 2006 or the 2013 NCHS Urban-Rural Classification Scheme for Counties

Calculation of the proportion of death

$$\text{Proportion of death} = \frac{\text{The number of deaths from a given cause in MM patients}}{\text{The number of deaths from all causes in MM patients}}$$

Calculation of the SMR

"CVD" refers to "#Diseases of heart", "#Essential hypertension and hypertensive renal disease", "#Cerebrovascular diseases", "#Atherosclerosis", "#Aortic aneurysm and dissection" and "Other diseases of arteries, arterioles and capillaries".

$$\text{CVD specific death rate} = \frac{\text{Total number of CVD deaths}}{\text{Total number of general population}}$$

$$\text{Person} - \text{years} = \frac{\sum(\text{The number of people for the same survival time} \times \text{Survival months})}{12}$$

$$\text{Expected death toll(E)} = \text{CVD specific death rate} \times \text{Person} - \text{years}$$

$$\text{SMR} = \frac{\text{The number of CVD deaths in MM patients(D)}}{\text{Expected death toll(E)}}$$

Calculation of the 95%CI (The value of $Z_{\alpha/2}$ is 1.96)

$$\mu_U = (D+1) \times \left(1 - \frac{1}{9(D+1)} + \frac{Z_{\alpha/2}}{3(D+1)^{1/2}}\right)^3$$

$$\text{SMR}_U = \frac{\mu_U}{E}$$

$$\mu_L = D \times \left(1 - \frac{1}{9D} - \frac{Z_{\alpha/2}}{3D^{1/2}}\right)^3$$

$$\text{SMR}_L = \frac{\mu_L}{E}$$

$$95\%\text{CI: } (\text{SMR}_L - \text{SMR}_U)$$

Calculation of the *P* value

$$z = \frac{\log_e \text{SMR}}{\frac{\log_e \text{SMR}_U - \log_e \text{SMR}_L}{2 \times 1.96}}$$

$$P = e^{(-0.717 \times |z| - 0.416 \times z^2)}$$

Supplementary legend

Table S1. Detailed disease clarification of each cause of death.

Table S2. Cardiovascular death-related standardized mortality ratios in different follow-up times.

Table S3. Cumulative mortality at follow-up time in patients overall.

Table S4. Cumulative mortality at follow-up time in patients diagnosed at age 85 or more years.

Table S5. Cumulative mortality at follow-up time in patients with localized disease.

Table S6. Cumulative mortality at follow-up time in patients with low-grade disease.

Table S7. Cumulative CVD mortality at follow-up time in different subgroups. 85^+ refers to age 85 or older.

Figure S1. Study design. Abbreviations: SEER, Surveillance, Epidemiology, and End Results.

Figure S2. Percentage of each cause of death in different malignant melanoma patient subgroups. (A)Patients with surgery treatment by stage group. (B) Patients without surgery treatment by stage group. (C) Patients with low-grade disease by stage group. (D) Patients with high-grade disease by stage group. (E) Male patients by age group. (F) Female patients by age group. (G) Patients with low-grade disease by age group. (H) Patients with high-grade disease by age group. (I) Patients with localized disease by age group. (J) Patients with regional disease by age group. (K) Patients with distant disease by age group. The height of the four different colored stacked bar charts showed the different proportion of deaths from malignant melanoma (black), other cancers death (dark gray), cardiovascular disease (light gray), and other non-cancer death (white), respectively. 85^+ refers to age 85 or older.

Figure S3. Percentage of each cause of death in different year of diagnosis. The height of the four different colored stacked bar charts showed the different proportion of deaths from malignant melanoma (black), other cancers death (dark gray), cardiovascular disease (light gray), and other non-cancer death (white), respectively.

Table S1. Detailed disease classification of each death cause.

Causes of death	Detailed disease classification
Malignant melanoma	Malignant melanoma
Other cancers	In situ, benign or unknown behavior neoplasm; Mesothelioma (ICD-10 only); Miscellaneous Malignant Cancer; Non-Melanoma Skin; Soft Tissue including Heart; Brain and Other Nervous System; Lung and Bronchus; Colon excluding Rectum; Esophagus; Intrahepatic Bile Duct; Liver; Pancreas; Rectum and Rectosigmoid Junction; Salivary Gland; Stomach; Kidney and Renal Pelvis; Prostate; Ureter; Acute Monocytic Leukemia; Acute Myeloid Leukemia; Bones and Joints; Chronic Lymphocytic Leukemia; Non-Hodgkin Lymphoma; Testis
Cardiovascular Disease	Aortic Aneurysm and Dissection; Atherosclerosis; Cerebrovascular Diseases; Diseases of Heart; Hypertension without Heart Disease; Other Diseases of Arteries, Arterioles, Capillaries
Infection	Other Infectious and Parasitic Diseases including HIV; Septicemia
Endocrine	Diabetes Mellitus
Nervous	Alzheimer's Disease (ICD-9 and 10 only)
Respiratory	Chronic Obstructive Pulmonary Disease and Allied Cond; Pneumonia and Influenza
Digestive	Chronic Liver Disease and Cirrhosis; Stomach and Duodenal Ulcers
Kidney	Nephritis, Nephrotic Syndrome and Nephrosis
Suicide, Accidents and Homicide	Suicide, Accidents and Homicide
Others	Symptoms, Signs and Ill-Defined Conditions; Congenital Anomalies; Other Cause of Death;

Abbreviations: ICD, international classification of diseases.

Table S2. Cardiovascular disease death-related standardized mortality ratios in different follow-up times.

Follow-up time (months)	SMR (95%CI)	*P* value [a]
1	19.91 (18.68–21.21)	< 0.001
2	6.41 (5.97–6.88)	< 0.001
3	4.04 (3.73–4.36)	< 0.001
4	3.02 (2.77–3.28)	< 0.001
5	2.57 (2.36–2.81)	< 0.001
6–8	1.94 (1.83–2.05)	< 0.001
9–11	1.49 (1.39–1.59)	< 0.001
12–14	1.29 (1.20–1.39)	< 0.001
15–17	1.08 (0.99–1.19)	0.08
18–20	0.96 (0.85–1.07)	0.43
21–23	0.91 (0.79–1.04)	0.17
24–26	0.81 (0.67–0.97)	0.02
27–29	0.66 (0.48–0.88)	0.01
30–32	0.55 (0.29–0.93)	0.04
33–35	0.38 (0.04–1.38)	0.28
36–38	0.29 (0.00–1.62)	0.43

Abbreviations: SMR, standardized mortality ratio; CI, confidence interval.

[a] Statistical significance was defined as $P < 0.05$.

Table S3. Cumulative mortality at follow-up time in patients overall.

Cause of death	Cumulative mortality (%)			
	5 years	10 years	20 years	30 years
Malignant melanoma	15.08	19.11	19.44	19.50
Other cancers	2.52	4.28	5.28	5.45
CVD	10.32	25.45	35.47	37.74
Other non-cancer	8.67	23.26	33.79	36.86

Abbreviations: CVD, cardiovascular disease.

Table S4. Cumulative mortality at follow-up time in patients diagnosed at age 85 or more years.

Cause of death	Cumulative mortality (%)			
	5 years	10 years	20 years	30 years
Malignant melanoma	16.11	17.21	17.29	NA
Other cancers	4.92	5.81	5.95	NA
CVD	26.18	37.71	41.65	NA
Other non-cancer	22.07	31.91	35.01	NA

Abbreviations: NA, not applicable; CVD, cardiovascular disease.

Table S5. Cumulative mortality at follow-up time in patients with localized disease.

Cause of death	Cumulative mortality (%)			
	5 years	10 years	20 years	30 years
Malignant melanoma	7.00	10.48	10.88	10.96
Other cancers	1.49	3.42	4.63	4.82
CVD	10.39	27.37	38.91	41.80
Other non-cancer	9.03	25.78	38.15	42.01

Abbreviations: CVD, cardiovascular disease.

Table S6. Cumulative mortality at follow-up time in patients with low-grade disease.

Cause of death	Cumulative mortality (%)			
	5 years	10 years	20 years	30 years
Malignant melanoma	13.78	18.52	18.52	NA
Other cancers	4.64	6.38	6.38	NA
CVD	9.08	23.03	27.05	NA
Other non-cancer	5.55	25.91	39.59	NA

Abbreviations: NA, not applicable; CVD, cardiovascular disease.

Table S7. Cumulative CVD mortality at follow-up time in different subgroups.

	Cumulative mortality (%)			
	5 years	10 years	20 years	30 years
Diagnostic age (y)				
65-74	4.55	9.71	25.04	35.63
75-84	12.31	22.99	36.03	NA
85^+	26.18	37.71	41.65	NA
Stage				
Localized	10.39	27.37	38.91	41.80
Regional	11.80	21.27	26.23	27.23
Distant	5.28	7.39	9.51	NA
Grade				
Low (Grade I+II)	9.08	23.03	27.05	NA
High (Grade III+IV)	9.11	20.28	26.77	NA

Abbreviations: NA, not applicable; CVD, cardiovascular disease. 85^+ refers to age 85 or older.

Figure S1. Study design.

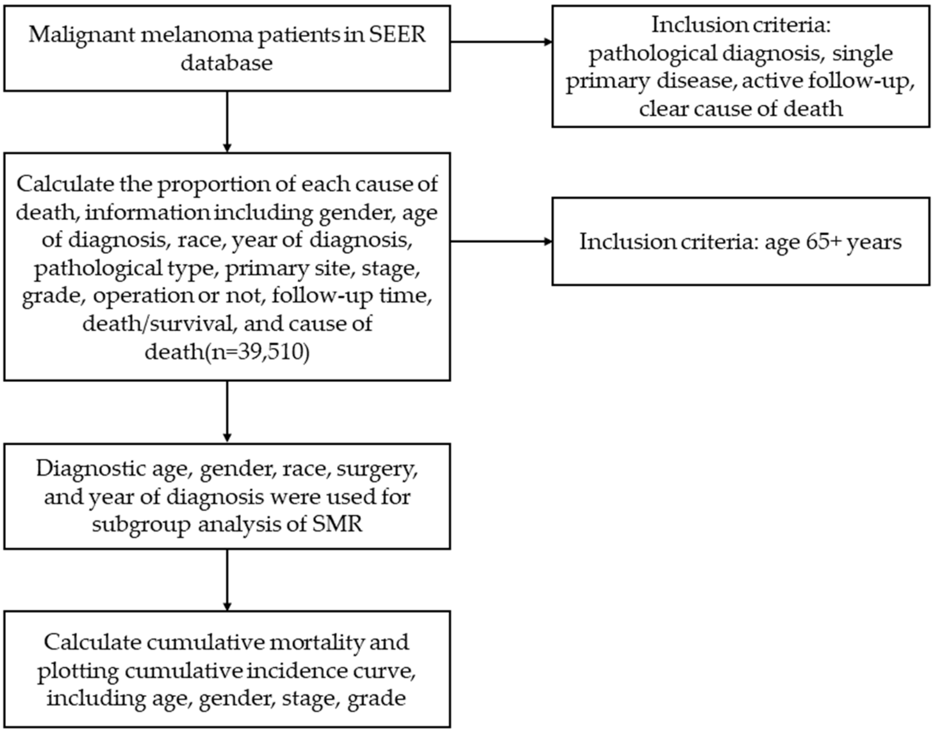

Abbreviations: SEER, Surveillance, Epidemiology, and End Results.

Figure S2. Percentage of each cause of death in different malignant melanoma patient subgroups.

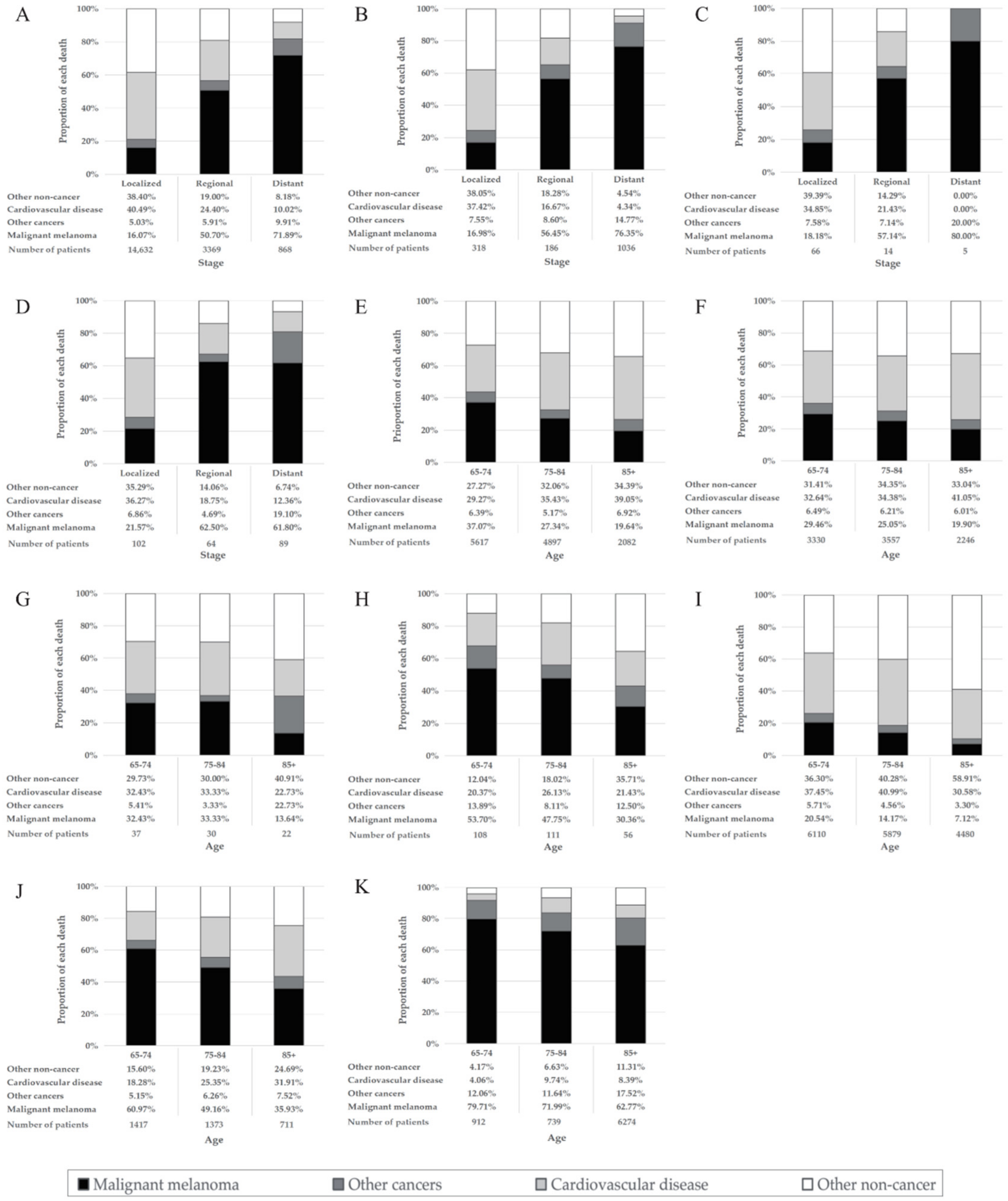

Patients with surgical treatment by stage group. (B) Patients without surgical treatment by stage group. (C) Patients with low-grade disease by stage group. (D) Patients with high-grade disease by stage group. (E) Male patients by age group. (F) Female patients by age group. (G) Patients with low-grade disease by age group. (H) Patients with high-grade disease by age group. (I) Patients with localized disease by age group. (J) Patients with regional disease by age group. (K) Patients with distant disease by age group. The height of the four different colored stacked bar charts showed the different proportion of deaths from malignant melanoma (black), other cancers death (dark gray), cardiovascular disease (light gray), and other non-cancer death (white), respectively. 85+ refers to age 85 or older.

Figure S3. Percentage of each cause of death in different year of diagnosis.

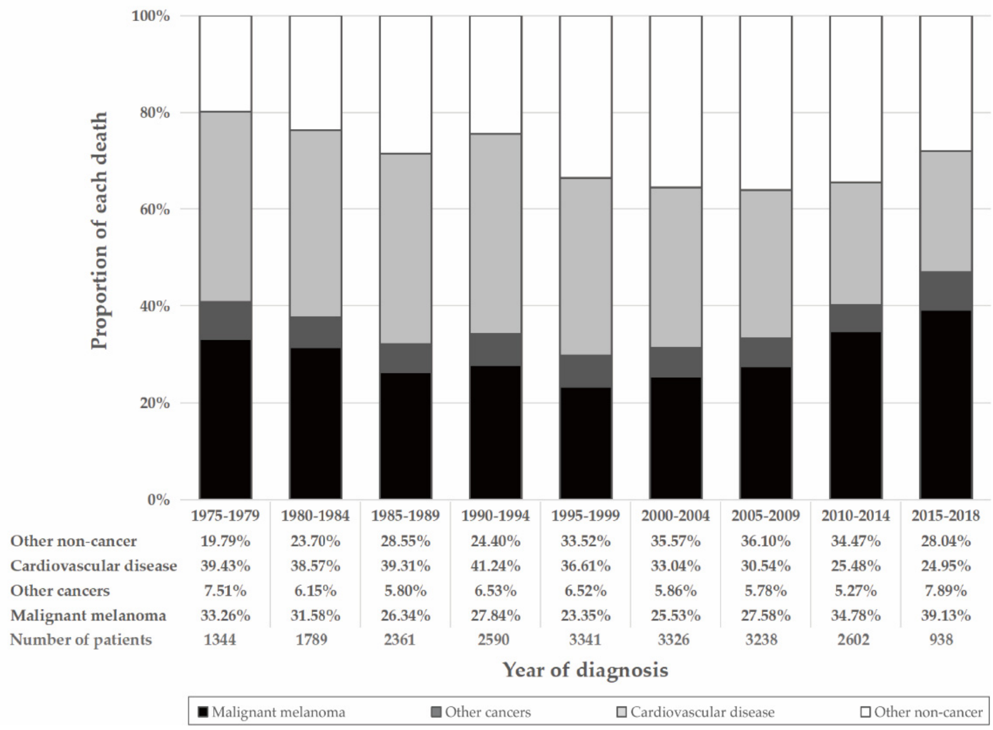

The height of the four different colored stacked bar charts showed the different proportion of deaths from malignant melanoma (black), other cancers death (dark gray), cardiovascular disease (light gray), and other non-cancer death (white), respectively.
